# Weight loss journeys: Exploring social influences and determinants of health in an exploratory rural German intervention

**DOI:** 10.1371/journal.pone.0330358

**Published:** 2025-08-14

**Authors:** Constanze Betz, Tina Bartelmeß

**Affiliations:** Faculty of Life Sciences: Food, Nutrition and Health, University of Bayreuth, Germany; Wits University: University of the Witwatersrand Johannesburg, SOUTH AFRICA

## Abstract

Overweight and obesity are highly prevalent across Western populations and contribute substantially to the global burden of non-communicable diseases such as cardiovascular disease and diabetes. Despite numerous intervention efforts, achieving and sustaining long-term weight loss remains a challenge. Social Determinants of Health (SDoH) play a critical role in shaping health outcomes **–** including weight and weight loss trajectories **–** and vary across social groups and geographic contexts. This qualitative study explores participants’ perceptions of personal and contextual SDoH influencing their weight loss journeys within a rural German population enrolled in a general practitioner-led pilot intervention. Semi-structured interviews were conducted with 16 participants from the HAPpEN trial (General Practitioner-led Obesity Prevention Program: Exercise and Nutrition), which focused on individuals living in a rural area of Upper Franconia. Interview data were analyzed using a grounded theory-informed approach to qualitative content analysis, guided by the SDoH framework as a sensitizing concept. Coding and theme development followed an iterative process involving constant comparison and discussion within the research team. Key determinants affecting weight loss efforts included individual routines and schedules, social and household networks, mental health, and work-related demands. Broader contextual factors such as socialization, cultural norms, and the availability of health-supporting infrastructure also emerged. The dynamic interplay between personal motivation and structural or environmental influences appeared to be central to weight management success. This study is limited by its small and relatively homogeneous sample – predominantly female and drawn from a single rural region – which may restrict transferability to more diverse populations or urban settings. Nonetheless, the findings offer practical implications for tailoring weight loss interventions to rural contexts by incorporating the complexity of individual and social determinants.

## Introduction

The prevalence of overweight and obesity among Western populations is significant [1] and recognized as a major risk factor for numerous diseases [2]. Despite frequent attempts at weight loss within these populations [3], long-term success rates remain low [4,5]. Clinical interventions and commercial weight loss programs typically focus on individual behavioral modifications such as dietary changes and increased physical activity [6]. However, these approaches often neglect the broader contextual factors that shape individuals’ lives. Obesity should not be viewed solely as a result of personal choices; it is influenced by a complex array of social determinants of health (SDoH) that affect an individual’s abilities to achieve and maintain healthier lifestyles [7,8].

The significance of these determinants – encompassing not only genetic predispositions and individual lifestyles but also environmental factors such as socioeconomic status, educational attainment, healthcare access, and community norms around nutrition and physical activity [9] – is insufficiently addressed in health research. According to Dahlgren and Whitehead [10], SDoH is a conceptual framework encompassing biological, social, and environmental influences on individuals’ health and well-being This model presents a layered, permeable structure ranging from personal to broader macro-level cultural factors The SDoH framework posits that the interplay of various factors predicts the individual possibilities for health and wellness [11], and when combined with additional measures, can be used to assess disease risk [12]. Importantly, the SDoH framework extends beyond merely considering socioeconomic status as an explanatory variable; it integrates social and cultural components often overlooked in the traditional analysis of overweight and obesity. While socioeconomic information is frequently employed to describe health disparities among populations or subgroups, it does not adequately measure the multifaceted determinants of health inequalities, as emphasized by Dahlgren and Whitehead [11]. Besides, there is a growing recognition that social variables play a pivotal role in the development and persistence of obesity [7,13]. However, the literature often conflates SDoH with socio-economic inequalities [14,15], a representation that diverges from the original intent of the SDoH framework. Research typically quantifies SDoH through parameters such as socio-economic status (SES) and demographic parameters to elucidate social risks [16–18]. As a result, cultural and social network factors such as family and neighborhood structure or community norms are frequently underrepresented in discussions surrounding SDoH in the context of obesity and weight loss. Furthermore, the perspectives of overweight and obese individuals regarding their environment and SDoH remain underexplored.

The literature on weight loss typically focuses on and distinguishes between motivators or facilitators and barriers to success regarding weight loss when considering environmental determinants [19]. Barriers often cited include a lack of motivation, logistical challenges, time constraints, and emotional factors such as stress, boredom, or sadness. Conversely, motivators are generally linked to body (dis)satisfaction, health concerns [20–22], as well as social support, appropriate exercise and diet regimes, and positive interactions with healthcare providers [23]. This body of research underscores the critical role of healthcare providers in directly motivating and supporting individuals or in offering guidance through their weight loss journeys [23,24].

Understanding patient perceptions of the factors that influence their health, such as their daily life structure and work environment, is essential for evaluating the efficacy of interventions. Although medical programs predominantly rely on clinical and behavioral guidelines, patients’ insights into the barriers and facilitators of their weight loss journeys are often overlooked. This oversight is particularly significant in rural areas, where social and environmental determinants may have a pronounced impact on lifestyle behaviors [25,26]. Rural areas are not only insufficiently researched in this context, but are also confronted with a different daily structure and different opportunities than urban areas, not only in terms of the built environment (e.g., lack of fitness facilities), but also in terms of low-threshold access to (specialized) healthcare [27,28]. There is a lack of research examining the perceptions of SDoH among rural German patients and their associations with weight loss. This study aims to address this gap by exploring the personal experiences and perceptions of participants of a weight loss intervention in rural Germany concerning the social determinants that affect their weight loss efforts, using qualitative semi-structured interviews to gather in-depth insights.

Gaining a deeper understanding of how these rural participants perceive the SDoH within the context of a GP-led weight loss intervention can provide invaluable insights to inform future intervention design. Additionally, these findings can guide policy and decision makers in identifying specific barriers to address, with the ultimate goal of pinpointing the most relevant social determinants that could facilitate effective and sustainable weight loss, thereby contributing to broader public health objectives.

## Methods

The HAPpEN exploratory trial is registered within the German Clinical Trials Register (DRKS00033916) and received ethical approval from the University of Bayreuth´s ethics committee (Az. O 1305/1 – GB). All participants had given written informed consent prior to the start of the study. The study protocol was previously published [29] and the data note is available at the open science framework (https://osf.io/8utdq/ and https://osf.io/5vzpy/).

In summary, this multicentered, general practitioner (GP)-focused exploratory trial incorporates a behavioral intervention targeting nutrition and physical activity, conducted both on-site at GPs’ offices and physiotherapist clinics and remotely via an app, over 12 months from 01/06/2023 to 31/05/2024. The trial was initiated on 15/04/2023 with open enrollment until 31/05/2023. The trial initially enrolled 112 eligible participants, all aged 18 years or older with a diagnosis of obesity (BMI ≥ 30 kg/m²) and excluded those with conditions preventing weight-bearing activities, severe contraindicating conditions, or diagnosed eating- or psychiatric disorders [29,30].

Participants received monthly appointments with their GP, physiotherapeutic group training sessions, and access to a digital study app with multiple tracking features, such as weight, heart rate, and blood pressure trackers, alongside a study website with platform access. Comprehensive health markers were assessed at the GP offices at baseline, 6 months, and 12 months.

In addition to quantitative data collection through measurements and standardized questionnaires within the HAPpEN trial, the study aimed to capture individual perspectives on SDoH at a more nuanced level. To this end, qualitative semi-structured interviews were conducted. Recruitment for these interviews started on 01/10/2023 and initially involved a digital invitation sent to all enrolled patients through the participants’ digital study diary, a mobile application that facilitated communication between the study team, medical staff, and participants. However, as no participants responded to the digital call, which was a push notification appearing daily for 30 days, including a call to action (‘If you’re interested, write an e-mail’), recruitment was supported by GPs, who informed participants about the interview option during their monthly check-ups. Interested individuals then scheduled interviews with the first author (CB), an experienced qualitative social science researcher.

Interviews were conducted at three time points during the study (27/10/2023; 17/11/2023; and 14/02/2024) in a private room at one of the GPs’ offices to ensure participants’ convenience and a neutral environment. Ultimately, 16 participants (22.8% of the remaining HAPpEN trial cohort of approximately 70 under open dropout conditions, i.e., participants could leave the study at any time) were interviewed between December 2023 and February 2024, corresponding to the intervention’s midpoint. All participants provided written informed consent prior to the start of the interview. The interview duration ranged from 8:05–30:21 minutes, with a mean of 15:23 minutes.

The semi-structured interviews followed a predefined interview guide (see Supporting Information S1 File A), were audio-recorded, and transcribed verbatim using the AI-based transcription service noscribe. Each transcript was subsequently reviewed and manually corrected by the research team to ensure accuracy and completeness. Interview questions were open-ended to facilitate in-depth discussion and to address the various dimensions of the social determinants of health model. All interviews were conducted by the first author (CB), a member of the research team not involved in direct patient care, to encourage candid and unbiased responses. In line with grounded theory methodology [31], sampling and analysis were conducted iteratively. Recruitment continued until the research team determined that informational redundancy and data sufficiency had been reached, meaning that no substantially new themes emerged in the final interviews, and additional data only yielded minor variations of previously identified themes. We acknowledge the ongoing and provisional nature of theme development in qualitative research, as recommended in recent methodological literature [32,33]. Thus, the sample size was determined pragmatically, based on the point at which further interviews contributed little new conceptual insight.

Interview data were analyzed using MAXQDA (2024, Verbi Software) through a qualitative content analysis informed by grounded theory principles [34,35]. An initial codebook was developed deductively based on the Social Determinants of Health (SDoH) framework [10,11] – commonly known as the ‘rainbow model’ – which served as a sensitizing concept and informed the design of the interview guide (see Supporting Information S2 Table B). This framework provided a conceptual starting point, encompassing categories ranging from genetic and individual lifestyle factors to social networks, living and working conditions, and broader environmental influences. In line with grounded theory methodology, the analytic process remained iterative and responsive to the data. The codebook was continuously refined during the analysis to allow for inductive identification of new themes, subcategories, and interrelationships not anticipated in the initial framework. This approach combined the deductive structure of the SDoH framework with grounded theory techniques such as constant comparison, memo-writing, and theory development grounded in the empirical material. Thus, the SDoH model functioned as an interpretive lens and ‘sensitizing concept’ rather than a prescriptive template [31], enabling the integration of existing theoretical knowledge with context-specific insights derived from participants’ lived experiences. This flexible and reflexive strategy aligns with contemporary applications of grounded theory in health research [36–38], facilitating both theoretical sensitivity and openness to emergent meaning.

The interviews were analyzed and coded independently but collaboratively by both authors. Transcripts were read repeatedly to ensure familiarization, and initial codes were generated by two researchers independently using an associative approach. Coding discrepancies were discussed and resolved in regular team meetings through verbal discussions and consensus, including peer debriefing sessions to critically reflect on emerging themes and minimize researcher bias. For example, there were differences in opinion regarding whether statements about lacking motivation after a stressful workday should be coded as ‘individual thought patterns’ (e.g., intrinsic will) or as ‘external determinant’ (e.g., motivation through social support and network). Similarly, some statements about ingrained eating habits formed during childhood were variably coded as ‘socialization’ or ‘psychological factors’. In each case, the team jointly reviewed the relevant transcript passages, discussed the underlying context and meaning, and agreed on the most appropriate code.

To ensure rigor, we adhered to established criteria of qualitative trustworthiness [39] – credibility, transferability, dependability, and confirmability – by employing methodological triangulation, member checking, and rich contextual descriptions. In line with qualitative research standards, we prioritized interpretive agreement and analytical depth over statistical measures of inter-coder reliability [33,40]. Peer debriefing sessions with team members not involved in the study were conducted by presenting interpretations within their context to test for researcher bias. Reflexive journaling and peer debriefing minimized potential researcher biases and ensured analytical transparency. To enhance transferability, we provided a detailed description of the research context, including participant demographics, relevant social characteristics, and the cultural and environmental setting in which the study was conducted. The sampling strategy and data collection procedures are systematically documented to ensure transparency. In the Results section, representative quotations are included to illustrate key themes and highlight contextual nuances. By embedding the analysis within its broader setting, we aim to support the transferability of findings to comparable contexts. These measures collectively strengthened the trustworthiness, consistency, and applicability of our findings.

In the interviews, participants, who are pseudonymized in chronological order, discussed the impact of social and environmental determinants on their weight loss efforts, offering personal insights into how factors such as social environments and networks, living and working conditions, and the broader context in a rural area in Germany influenced their ability to engage with and sustain behavioral changes.

## Results

### Participants characteristics

Among the 16 interview participants, the majority were female (14/16) and over the age of 40 (12/16). A detailed overview of basic characteristics can be found in Table 1. Most were employed or engaged in care work, while two participants were retired and not involved in care work. Although a more diverse sample was sought, low response rates limited the diversity of participants. The final sample of 87,5% females within this study might limit gender generalization, but is consistent with the predominantly female population within weight loss interventions [41]. Furthermore, this gender distribution within our interview sample is consistent with the overall participant characteristics of the HAPpEN trial, where approximately 74.1% of participants were female [29]. Additionally, the interview sample was relatively homogeneous compared to the overall HAPpEN trial population, which is well-aligned with the principles of grounded theory, facilitating in-depth exploration within a focused participant group [33].

**Table 1:** Participant characteristics (n = 16).

Characteristic	n	Participants (%)
**Sex**		
Female	14	87.5%
Male	2	12.5%
**Age Group**		
18–29 years	2	12.5%
30–39 years	0	0.0%
40–49 years	4	25.0%
50–59 years	6	37.5%
60 + years	4	25.0%
**Employment Status**		
Employed or care work	14	87.5%
Retired and not in care work	2	12.5%

*Percentages refer to the total number of participants. ‘Care work’ includes unpaid caregiving responsibilities such as caring for children, elderly family members, or others in need of support.*

Participants in the HAPpEN trial generally reported long-persisting histories of being overweight, often dating back to childhood. Nearly all participants reported multiple previous weight loss attempts, with many having over a decade of experience. Further information is published elsewhere [29,42].

Key themes that emerged during individual interviews included individual lifestyle factors and social network support, mentioned by almost all participants (15/16). Work-related conditions were the most significant among broader life and work factors, cited by 12 out of 16 participants. Notably, broader factors such as genetics and cultural, socioeconomic, and physical environment factors were infrequently mentioned.

### Social influences and determinants of health

Instead of organizing the themes by frequency or perceived importance, we structured the presentation of results according to the layered logic of the SDoH framework, which arranges factors from individual to broader societal influences. Table 2 summarizes the central themes identified through the interviews, highlights key findings within each thematic area, and indicates whether the observations are generalizable or show rural/urban-specific patterns.

**Table 2:** Overview of main themes, key findings, and contextual distinctions (rural, urban, general).

Topic	Key Findings	Context (Rural/ Urban/ General)
**Genetics & Personal Health**	● Frequent mention of chronic health issues (e.g., diabetes) ● Mixed perceptions of pharmaceuticals (side effects vs. benefits) ● Skepticism toward generalized dietary advice; preference for individualized recommendations	General
**Individual Lifestyle Factors**	● Strong impact of work schedules, childcare, meal timing, and daily routines ● Adaptive behaviors: skipping meals, time-restricted eating, reduced prioritization of physical activity ● Program enabled conscious food-related discussions within households	General **Rural-specific:** long commute distances; scheduling conflicts due to childcare and routines
**Socialization, Upbringing & Habitualness**	● Deeply ingrained eating habits shaped by post-war generational food culture ● Lasting influence of overfeeding, strict food control, and food taboos in childhood ● Emotional and secretive eating; self-regulation difficulties ● Mental health and motivation are frequently mentioned ● Some adopted coping strategies (e.g., walking, social support)	General, with rural lean (cultural generational influences, rural behavior patterns)
**Social Networks & Support**	● Household and peer support were crucial for success ● Unsupportive partners or mental illness in close others created barriers ● Motivation was boosted through peer encouragement (e.g., walking groups, physiotherapists) ● Perceived credibility of advice influenced acceptance (e.g., success stories vs. “slim” advisors) ● Emotional support improved adherence; its absence led to dropout	Primarily rural (closer-knit community dynamics)
**Living and Working Conditions**	● Major barriers: work overload, time constraints due to dual responsibilities (work and home) ● Shift work and long hours limited opportunities for meal prep and physical activity ● **Rural-specific:** limited access to plant-based food; cultural preference for high-fat, meat-heavy diets ● Positive exception: workplace health initiatives	Both with rural emphasis (dietary culture, food availability)
**Environmental & Cultural Factors**	● Isolated mentions of the physical environment (positive: forest access; negative: unsafe roads) ● Societal expectations and local norms influenced behavior (e.g., social pressure to attend food-centric events)	Mostly rural (village norms, local traditions)

#### Genetics.

Genetics, as well as personal health concerns, were discussed infrequently, with only one participant directly referencing genetics. However, five of the 16 participants noted the role of personal health, including conditions such as diabetes, in their weight management efforts. Some participants expressed skepticism about the role of pharmaceuticals, particularly concerning weight loss, citing negative side effects like reduced resilience. However, others acknowledged the positive impact of medications that allowed for increased physical activity. Two participants criticized the oversimplified view of food as strictly ‘good’ or ‘bad’, advocating for individualization in dietary advice.

*“And I think you also must find out, you can’t generalize, switch to whole grain products. Eh. Whole grain products - yes. It works for some, but some people gain weight on whole grain products.” (t1)*

#### Individual lifestyle.

The theme of individual lifestyle encompasses not just personal choices but also the influence of upbringing, social context, living and working conditions, as well as cultural and societal influences, aligning with Dahlgren & Whitehead’s [11] view that lifestyle is shaped by multiple external determinants. Participants highlighted the impact of work types and schedules, household routines and division of labor, childcare responsibilities, meal preparation, and other daily chores on their eating and physical activity habits. For example, meal planning was often shaped by children’s extracurricular activities. Some participants skipped meals due to their work schedules, time constraints, or personal meal timing, such as the 16−8 schedule, or reported eating while engaged in other tasks. Similarly, fitting physical activity into a busy schedule, especially group-based activities was challenging due to time constraints or lack of motivation after fulfilling work and household responsibilities.

*“So, stress at home, kids at school, homework, household, and work too. So, it’s quite stressful now and then you just don’t feel like doing anything in the evening.” (t2)*

Meal preparation habits were largely dictated by household dynamics, with negotiations between family members playing a crucial role in meal planning. Participants described how the HAPpEN trial influenced household discussions around food, leading to healthier choices in some cases.

*“It helps people (..) to live and eat healthier. We’ve never thought so much about what needs to be cooked now and why. Especially the plate thing. A half, a quarter, a quarter. We discussed it. Is the salad enough now?” (t13)*

#### External determinants: Socialization, upbringing and habitualness.

In contrast, a few participants noted that external determinants such as long-standing habits, formed during their socialization and upbringing, persisted despite their awareness of healthier alternatives. Many participants also reported difficulties in consistently maintaining new habits due to ingrained routines and the influence of social and familial expectations. Participants’ narratives around upbringing were shaped by generational experiences, particularly among those raised by post-World War II survivors in Germany. These experiences contributed to attitudes toward food, with some participants recounting being ‘overfed’ as children. This upbringing, characterized by high-fat and high-sugar diets, continues to influence participants’ eating habits and attitudes toward food.

*“My husband habitually makes vegetable soup with meat, and I explicitly have to tell him not to - just like with frying, he’s raised like this, and I have to tell him to use oil, because... of course, he’s a generation, so we both stem from a generation, belonging to our parents as Post-War children. Of course, everything had to be very high in fat; we grew up like that, no. So, the fatter, the better, because we had to have something to put on, that’s what they used to say. And of course, we struggled with that too.” (t1)*

Younger participants also reflected on how they were raised with rigid dietary controls, which often led to secretive eating habits and poor self-regulation around food. For several participants, these early experiences still resonate in their current eating patterns and struggles with food.

*“And I’m saying in my youth. I’m not that old yet - my mum looked at it very, very, very closely back then. I’ve also been to rehab twice, which ultimately didn’t really help, because afterward I was back on it again, and that’s when I had a problem with snacking. The snack cupboard was locked, and the key was gone. That was just so hmm. And it’s really dragged on until now that I can’t stop at five jellies, but instead eat the whole bag, to put it bluntly, because I didn’t learn it back then, I’ll say it now, but it was a taboo subject for me. You’re too fat anyway, and then it’s just a bit of a drag.” (t4)*

Participants consistently reported that their current eating habits were strongly influenced by early life experiences, whether through a lack of learned self-regulation or the internalization of specific dietary patterns, such as the perceived necessity of consuming a certain amount of meat per week. While all participants acknowledged at least minor biographical factors shaping their eating behaviors, for a significant subset, these habits persist as coping mechanisms or are associated with emotionally charged memories of food-related taboos.

“*It’s always been an issue. It’s always been, yes, it’s always been a matter of not eating too much or something like that. Or you just ate in secret. (..) But it was always so difficult because my father was sometimes extreme. I know that he sometimes took my mum’s plate away, told her to stop eating, and did stupid things like that. But yes. (...) It was always a difficult subject” (t14)*

Psychological factors, including the emotional impact of food, also emerged as a significant theme. Nine participants discussed the mental health challenges associated with maintaining motivation for weight loss and resisting temptations. Some described strategies for overcoming these challenges, such as seeking social support, while others found it difficult to break old habits or maintain self-discipline.

*“But it’s also really a lot of personal responsibility. (...) Probably only a chip in my head really helps. (..) Yes, that’s the case. These are things that you know, you know that. I can’t imagine that there’s a fat person who doesn’t know everything that’s been said. I can’t imagine that. (laughs) Who has never heard of calorie deficit? Or something like that. (...)” (t9)*

Participants identified mental health challenges, both personal and those affecting close others, as significant barriers to behavioral change. They unanimously agreed that mental health and overall well-being had a substantial impact on their eating behaviors, general well-being, and motivation. Coping strategies varied considerably; while some individuals engaged in ‘mental health walks’ or ‘grabbed a friend for support’, others responded to distress by withdrawing or remaining indoors.

Most participants agreed that unlearning ingrained behaviors posed a significant challenge (e.g., *“I’ve been snacking on sweets every night for 40 years, so of course it will take 40 years to unlearn that.” [t6]*). However, some participants reported successfully adopting new habits, emphasizing that awareness of these behaviors is critical for change.

*“So that’s my problem. Learning to endure and looking for alternatives when there’s no other way. At work, I have the alternative right now, instead of reaching into my colleagues’ sweet pot, there are now walnuts in my office. Well, it’s time for walnuts again, right, and then... or mandarins. So that’s my favorite now. Anything that I must cut up quickly or something, not so much. Or the fruit box, where you say I must force myself to cut fruit consistently every morning. (t1).*

#### Social network and support.

Social networks, particularly the influence of household members, were found to play a critical role in participants’ ability to adhere to the weight loss program. Both positive and negative social influences were identified. Some participants reported encountering unsupportive attitudes from family members or partners, particularly when those individuals were experiencing their own mental or physical health challenges, such as depression. Additionally, concerns about social control and the fear of being judged for not conforming to social norms – such as refraining from consuming cake or alcohol at social events – emerged as a significant barrier to weight loss. This perceived pressure to align with societal expectations was recognized as an obstacle to sustained behavioral change.

In a few instances, participants described unique social network circumstances that impeded their ability to focus on weight management. Specifically, recent family bereavements were cited as having a profound effect on their capacity to prioritize weight loss. Moreover, participants acknowledged that the acute stress stemming from these events contributed to emotional eating as a coping mechanism. As one participant described, *“This is a factor that leads to me sitting in the evening and craving half a bag of chips...” (t10)*.

In contrast, many participants described how family members and other social networks, such as those established through the GP practices outside of the group-based interventions or by the physiotherapists, motivated them by often providing companionship during physical activities or offering encouragement during the program.

*“My husband and my son, by having dinner with me, and when I say I don’t feel like going out, then my husband says, come on, you go with me now. So, they support me a lot!” (t10)*

*“No, I definitely feel supported because we always meet now and go together. We all motivate each other.” (t14)*

Overall, the presence of supportive individuals within participants’ social networks emerged as a key factor in their ability to consistently implement the recommendations of the HAPpEN program. Participants who lacked a sense of community reported greater difficulty in adhering to the program´s guidelines. Occasionally, the social environment was also mentioned as a source of role modeling and practical advice. However, the perceived credibility of this advice was contingent on the experience of the individual providing it. Participants valued guidance from those who had successfully lost weight over advice from those who had not.

*“Yes, my friends try to help me, but it’s rather difficult. But that’s up to me. If someone who has 90-60-90 tells me to look like this, it comes across as a bit implausible... if someone who has already lost 52 kilos says hey, that helped me, you can try that, then that’s something else.” (t4)*

Participants also responded positively to input from physiotherapists, whose insights were seen as expert advice delivered without an overly didactic tone:

*“I’m also independent of HAPpEN in several physiotherapy practices and what they say is completely in the direction of HAPpEN, without them knowing about it. And it strikes me that they clearly follow this line, of course, they know the internal and external connections. They see the goal for themselves and how it can be achieved and know the meaning of what it is good for. And then you can ask questions, they just explain it to you, they simply give answers. And the connections are in the foreground.” (t13)*

However, obtaining support from one’s social network was often dependent on effective communication. Participants who did not prioritize diet and exercise tended to avoid seeking support, which contributed to a perceived lack of social reinforcement. Additionally, many found it challenging to follow externally prescribed routines, such as physiotherapy exercises that required self-motivation or engaging in activities like walking, which were favored by others in the social network but not personally enjoyed.

#### Living and working conditions.

The theme of living conditions encompasses several factors that appear to be regarded as less significant by participants in the HAPpEN trial, when compared with the Dahlgren & Whitehead model, likely due to the specific cultural and socioeconomic context of the study population. Elements such as water quality, sanitation, and housing conditions were neither considered nor mentioned by the participants. Furthermore, the healthcare system was not identified as an area of concern. While food infrastructure was mentioned only once, it was generally perceived as adequate. However, access to healthier, plant-based food options was considered limited in rural Upper Franconia, where the traditional diet is characterized by a higher intake of meat and fats, with less emphasis on vegetables and other healthier alternatives.

In contrast, work and working conditions emerged as one of the most influential environmental determinants impacting participants’ ability to adhere to the program, particularly by affecting their schedules and energy levels. This was not only limited to the proportion of shift-workers, who face additional challenges such as an often-changing schedule or matching their free time with their environments, but also regular employees.

*“So, 60 hours a week, it’s clear that after that I don’t feel like doing HAPpEN (...). (...) But at work it’s just work. (...) So I try to fit it in by going to the canteen. I mean, I must walk five to six hundred meters to get there. If I can’t do that at work, for example, then I won’t achieve my walking goal. So, it was also much better in the summer (...) because you don’t have so much stress at work because we had more staff.” (t6)*

Participants reported that the demands of balancing work with household responsibilities often constrained their time for physical activity and meal planning. This challenge was not limited to individuals directly engaged in employment but extended to all participants, as work-related conditions influenced household dynamics. The detailed impact of work on individual schedules, as previously discussed, further highlights the interconnectedness of various social determinants and their cumulative effect on lifestyle and health behaviors.

*“I leave the house at 7 a.m. and don’t get home from work until half past six in the evening. So, there’s not much in the way of leisure activities, HAPpEN and so on because you’re just tired in the evening. And at some point, that’s enough. And at the weekend I must get the household in order or keep it running.” (t13)*

Worksites, on the other hand, can be a beneficial environment if worksite health promotion is applied. One participant mentioned his company’s progressive initiatives regarding lunch options and health-promoting education that were nevertheless highly dependent on the company’s leadership.

*“I can choose how I need it at our canteen. You can combine everything with your individual needs, or leave things out. They [the company] gave many inputs, for example education regarding sugars and blood sugar regulation. We had a campaign called brainfood and the lunch coma was made a topic at events. But sadly, since our mastermind in the works council is no longer with us, everything has gone back to sleep.” (t6)*

#### General environmental factors.

Participants seldom consider broader environmental or cultural factors as significant influences on their health. Only two participants mentioned their physical environment, with one appreciating the local forest for cycling and another expressing concerns about road safety. Cultural influences, including societal norms around food and body image, were minimally discussed, although a few participants acknowledged the impact of societal expectations on their health behaviors.

*“Especially in the village, this social (...). You look at each other, it can be very enveloping, but it can also be very oppressive. (...) And then social norms, yes, that’s the way it should be. For example, there’s a theatre night here. I don’t feel like going, okay, but I just must go. (...) You just sit there, which is not good for my health. Then I can say I hope there’s water to drink. But I don’t order food there. But it’s usually the case that the clubs make money from the food.” (t3)*

In summary, while individual lifestyle, social support, and work conditions were key determinants of participants’ health behaviors, broader environmental and cultural factors played a less prominent role in shaping their experiences with weight loss. The interconnection between personal choices, social networks, and working conditions highlights the complexity of addressing weight loss within the context of the HAPpEN trial.

## Discussion

Despite high engagement in weight loss activities, long-term success in maintaining reduced weight is rare but possible, as demonstrated in this and other studies [4,43]. Although most clinical trials and commercial programs prioritize behavioral interventions (e.g., dietary modifications and increased physical activity), the HAPpEN trial stands out by fostering a social component that encourages participants to connect with others who share similar weight loss goals. This social connection may prove pivotal in sustaining motivation and adherence, highlighting a potential avenue for future research in weight loss and maintenance interventions. Consequently, future programs should incorporate structured peer networks, group meetings, or buddy systems to promote sustained engagement.

The study reveals that on an individual level, time constraints significantly impact the ability to maintain weight loss efforts. Employed adults, particularly those in rural settings, often struggle to allocate time for health-promoting activities due to busy schedules and long travel distances. These challenges limit the flexibility needed to incorporate exercise or meal preparation into daily routines. The rural lifestyle also often includes rigid, traditional schedules, such as fixed mealtimes, which may reinforce patterns that are less conducive to adaptive lifestyle changes. While these schedules might complicate health-promoting behaviors, designing interventions modular and adaptable might be beneficial. Such modular and adaptable interventions could, for example, use digital tools, asynchronous program elements, and incorporate the built environment.

Notably, while routines in the rural lifestyle can facilitate shared household activities (e.g., shared meals or discussions on health and fitness), they may also create burdens. For example, family members who support one another in managing daily life may feel the weight of additional lifestyle changes. These complex dynamics underscore how structured daily routines and family roles can both support and inhibit health-promoting behaviors and enhance prior research on social dynamics in weight loss and maintenance context [41,44–47]. Thus, social-support-based, family-inclusive program elements that account for collective behavior change and shared responsibilities might be helpful, for example, by including family-based counseling or meal planning tools that allow collaborative goal setting within household structures [48].

Interestingly, the study found that participant engagement in weight loss efforts was often tied to their perception of social support, which, in turn, appeared influenced by individual attitudes toward health topics. Participants who showed greater interest or openness toward nutrition and exercise were more likely to perceive their social environment as supportive, whereas those less inclined toward these topics often reported feeling isolated or withdrawn from the program. This indicates that personal motivation can play a crucial role in engaging with weight loss efforts and in interpreting social feedback as either supportive or obstructive. Additionally, participants who involved their households in lifestyle changes experienced this as positive reinforcement. When household members were supportive of new routines, particularly in terms of dietary choices and physical activities, participants reported higher satisfaction and commitment to the intervention. Conversely, unsupportive partners or environments where weight loss was trivialized posed obstacles to sustaining these changes, reflecting findings from prior research on the importance of positive social relationships in fostering healthy behaviors [20,49–51]. Building on existing evidence that community context influences health behavior in weight loss efforts [41,44–47], recent research further highlights the role of both immediate and extended social networks – and the nature of their support or sentiment – in shaping individual weight management outcomes [45,52–54].Regarding intervention design, this reinforces the importance of addressing both interpersonal and intrapersonal dimensions in intervention design. Personalized motivational interviewing, coupled with social network mapping and partner or family engagement strategies, could help tailor support mechanisms based on individual needs and social embeddedness.

Positive social influences are context-dependent, and support within close relationships varies widely. For instance, studies show that a spouse can either bolster or inhibit weight loss efforts depending on their attitudes and behaviors [51]. This variation supports the argument for group-based interventions, which connect individuals with shared goals, allowing participants to find relatable role models and fostering social modeling [55,56]. Literature on nutrition communication aligns with this perspective, showing that shared weight loss journeys—whether through in-person dyads or virtual platforms like social media—can enable participants to identify with and learn from peers who have faced similar challenges [57]. Studies, such as Arigo [58], affirm the efficacy of dyadic approaches in promoting adherence and motivation in weight management efforts.

Additionally, work environments and conditions were identified as influential factors in weight loss efforts. Work schedules not only shape social networks within and outside the workplace but also impact lifestyle opportunities [59]. Participants reported that shift work hindered participation in program activities, including weekly walking sessions and group physiotherapy. Work schedules also influenced household routines, such as meal timing and available time for exercise. These findings underscore that those determinants beyond individual control, such as workplace structures, can profoundly impact personal lifestyle choices and health behaviors and must be considered in intervention design [60,61]. Given the substantial time spent in the workplace, participants emphasized the value of health-promoting workplace initiatives. Examples included healthy workplace canteens offering nutritious meals and communal cooking opportunities, which were perceived as supportive. However, the continuation of such programs was often dependent on managerial support, with some participants expressing disappointment when these initiatives were discontinued following leadership changes. As a consequence, interventions could account for these environmental limitations, possibly through community-based food access initiatives, mobile markets, or partnerships with local suppliers [57].

Furthermore, cultural aspects tied to rural and urban distinctions emerged as critical. Rural areas, often characterized as ‘food deserts’ for certain types of food [62], lack access to diverse, nutritious options compared to cosmopolitan urban regions. Additionally, rural family life tends to adhere to traditional social patterns and norms that shape daily schedules, meal timing, and health behaviors, sometimes creating barriers to health management [63]. This adherence to tradition reflects ingrained norms that shape individual choices and socialization, emphasizing the influence of deeply rooted, intergenerational habits on health behaviors. Although the SDoH model by Dahlgren and Whitehead [10] includes cultural factors as determinants, it does not fully capture these habitualized norms that emerge from generational patterns and impact individual health agency.

Social norms and expectations around food and lifestyle in rural communities often limit flexibility in health-related behaviors, especially since deviations from the norm are more noticeable in tightly knit social environments [64]. In urban settings, the relative anonymity of residents affords greater freedom from social scrutiny, whereas in rural areas, perceived community expectations may act as a constraint on individual health choices. This narrower ‘room for maneuver’ means that health-promoting interventions must be tailored to consider both the social cohesion and the cultural rigidity that may exist in rural communities, to create effective and lasting change [65,66]. Culturally sensitive messaging, the inclusion of local community leaders, and normalizing change through group identity (e.g., community-wide health challenges) could be effective.

Additionally, the contrast between rural and urban social dynamics has critical implications. In rural areas, social pressure can deter health behaviors that diverge from community norms, while in urban areas, anonymity may facilitate experimentation and autonomy. This suggests the need for differentiated intervention models by setting: rural interventions should leverage strong social ties through community coalitions, while urban programs might focus more on individual autonomy, digital platforms, and anonymity-friendly support systems [59–61].

In summary, this study provides important insights for tailoring weight loss interventions to the rural context. Programs should prioritize:

- Time-flexible and modular program delivery to accommodate rural work-life patterns.
- Family- and household-oriented components that align with entrenched social routines.
- Sustained workplace health promotion backed by policy, not individual leadership.
- Culturally sensitive design that respects rural norms while encouraging adaptive change.
- Community-driven support structures to reduce isolation and normalize new behaviors.

By addressing the unique interplay of social, cultural, and structural determinants in rural settings, future interventions can be more effective in supporting sustainable weight loss and health behavior change in underserved populations.

## Limitations and further research

This study investigates SDoH in a rural German population, specifically examining how social networks and support systems within the HAPpEN program foster health-related behaviors. The emphasis on social components aligns with SDoH frameworks, which underscore the importance of community and support in shaping health behaviors. However, the SDoH framework, while informative, has limitations. It is a broad tool designed to help identify target areas for health promotion, but it may not address nuanced needs across diverse population subgroups, especially among rural populations.

The qualitative interviews provided in-depth insights into participants’ views, reinforcing that both immediate and extended social environments strongly influence weight management behaviors. Many participants traced weight issues back to childhood socialization and early life experiences, indicating that these formative influences often remain unaddressed in current interventions, including HAPpEN. Recognizing socialization as a foundation for weight-related behaviors could present new avenues for targeted research.

Although the study’s qualitative design and small sample size limit generalizability, the findings are consistent with established literature. However, the predominantly female sample may not adequately reflect male experiences with rural weight loss, potentially limiting the applicability of findings across genders. Recruitment from a single GP practice within one region of Upper Franconia constrains geographic diversity and, therefore, the transferability of insights to other rural settings with different cultural, economic, or infrastructural characteristics. Moreover, participants were already engaged in the structured HAPpEN intervention, suggesting they may represent a more health-conscious or motivated subgroup, which could skew perceptions of feasibility and adherence. Taken together, these relatively homogeneous sample characteristics reduce the transferability of findings to broader or more diverse rural populations, where gender balance, levels of motivation, and access to structured support may differ substantially.

Future research should explore the dynamics between limited social support and individual disinterest in weight loss and evaluate methods for creating supportive groups that promote sustained health behaviors. Additionally, it would be beneficial to investigate the specific impact of rural life and work conditions—such as work profiles, daily commute, and distance from workplaces—on adherence to weight loss programs and overall lifestyle outcomes. Future research should further explore the integration of health-promoting workplace initiatives within existing policy frameworks to better understand their feasibility and impact. Additionally, comparative studies examining similar interventions in different national contexts could provide valuable insights for policy development and implementation.

## Supporting information

S1 File A – Interview guide. (DOCX)

S2 Table B – Coding manual. (DOCX)
