# Supplementary material for: Weight loss journeys: Exploring social influences and determinants of health in an exploratory rural German intervention
Source: PLoS One. 2025 Aug 14;20(8):e0330358. doi: 10.1371/journal.pone.0330358 (PMC12352671; doi:10.1371/journal.pone.0330358)
Supplement: S1 File — (DOCX) [file pone.0330358.s001.docx]

Supporting Information

## Weight Loss Journeys: Exploring Social Influences and Determinants of Health in an Exploratory Rural German Intervention

## A – Interview Guide

*The interview will be narrative (semi-structured) to support the participant’s natural flow of speech. The goal is to identify the types of relationships that have a positive or negative impact on participants’ subjectively perceived success in the HAPpEN intervention study, with regard to their exercise and dietary behaviours.*

**Preparation**

**Opening Statement:**
Thank you in advance for taking the time and being willing to participate in this interview. I would like to record our conversation and take a few notes. Would that be okay with you?

**Action:**
Read the consent form aloud.
Then: *Start recording*

**Introduction**

Before we begin, I’d like to say a few introductory words:
HAPpEN primarily focuses on improving obesity prevention and treatment in rural areas. However, we’re also interested in how your environment and personal relationships play a role.
During the interview, I’ll ask you various questions—you can respond with whatever comes to mind. There are no right or wrong answers. Please just share your honest thoughts.

**Prompt:**
Do you have any general questions before we begin?

**Social Environment**

1. ***General Perception***

**Question:**
To start with, could you please describe your social environment?

*(The social environment refers to the people with whom you have regular contact.)*

**Follow-up Prompts:**

- Think about your everyday life: which groups of people play a role in it?
- You can also describe what a typical day looks like.
- And what about on weekends?

1. ***Context***

**Question:**
What is your contact with these people like?
How often do you interact? Is it more intensive or more casual?
What kinds of things do you do together? In what *contexts* do you meet?

**Follow-up Prompts:**

- For example, do you have weekly get-togethers with friends or acquaintances?
- What does your partner/spouse do?
- How is your social life structured?

1. ***Relationship Dynamics***

**Question:**
Who usually initiates these contacts?
For example, with your neighbour, do you greet them first, or do they initiate?
Do you always call your friend, or do you take turns?

**Follow-up Prompts:**

- And with [specific person or group], how is it?
- Does your spouse/partner initiate activities? Or is that your role? Is it the same for other topics as well?

**Influence of the Social Environment**

1. ***Perception***

**Question:**
Thinking about the people you’ve just described: who among them influences your eating or physical activity behaviour?

**Follow-up Prompts:**

- Do you discuss HAPpEN or your health habits with any of them?
- Who is (jointly) responsible for food in your household?
- Who decides how your free time is spent? And what does that look like?

1. ***Context***

**Question:**
In what situations or contexts do these people influence your eating or physical activity behaviours?

**Follow-up Prompt:**

- Think about your daily routine—when does this tend to happen?

1. ***Relationship Dynamics***

**Question:**
To what extent do you bring up topics like nutrition or exercise in these relationships?
And to what extent do the others bring up these topics with you?

**Follow-up Prompts:**

- How often do you talk about nutrition or exercise with the people you've mentioned?
- How much are these topics discussed in your broader environment?

**Classification of Influence**

1. ***Perception***

**Question:**
Can you think of reasons why the people you've mentioned influence your eating or exercise behaviour?
(For example: Your spouse is involved in cooking and wants to have input on meals.)

**Follow-up Prompt:**

- To what extent do you feel supported or hindered by these people?

1. ***Context***

**Question:**
Can you describe situations where you feel particularly supported—or hindered—by someone?

**Follow-up Prompts:**

- To what extent is this related to habits or routines?
- How might the setting influence this? For instance, how would it be different if you lived in a city, or were on vacation?

1. **Relationship Dynamics**

**Question:**
How does your relationship with that person affect your experience of influence?
(For example, in the case of neighbours: Do you fear gossip or social stigma?)

**Closure**

**Statement:**
Thank you very much for the interview. That’s all from my side.

**Prompt:**
Is there anything else you would like to share on this topic?
