# Supplementary material for: Weight loss journeys: Exploring social influences and determinants of health in an exploratory rural German intervention
Source: PLoS One. 2025 Aug 14;20(8):e0330358. doi: 10.1371/journal.pone.0330358 (PMC12352671; doi:10.1371/journal.pone.0330358)
Supplement: S2 Table — (DOCX) [file pone.0330358.s002.docx]

Supporting Information

## Weight Loss Journeys: Exploring Social Influences and Determinants of Health in an Exploratory Rural German Intervention

## B - Coding Manual

This supplementary material provides an overview of the coding framework used in the analysis. The tables below include definitions, translated anchor examples, and coding rules for each main category.

**Please note:** All participant quotes were originally collected in German and have been translated into English for the purpose of this publication.

Table S1: Overview of Main Categories in the Coding Manual

| **Main Category** | **Table Reference** |
| --- | --- |
| Personal narratives (core beliefs) | S2 |
| External Determinants | S3 |
| Relational Direction | S4 |
| Network Relation | S5 |

Table S2: Coding Manual – **Personal narratives (core beliefs)**

| **Subcategory** | **Definition (Statements by participants that address…)** | **Anchor Example** | **Coding Rule** |
| --- | --- | --- | --- |
| **Individual Thought Patterns** | Individual, often unconscious thought patterns that may guide thinking or decision-making. | — | Applies to all subcategories: distinguish from *determinants*. Thought patterns refer to internal explanations (e.g., *“I'm too sick to be active”* – thought pattern) vs. external determinants (*“I had surgery last week”* – factual, outside reason). |
| **Comparison** | Perception that comparison plays a role in current or desired eating or physical activity behaviour. | “So, my colleague, for example, she frustrates me because she utilized the start to take care of herself and now, she has lost 42 kg, and I lost only 7.” (T4, Pos. 23) | Distinguish from social control and expectations from others. |
| **Personal Health** | Perception that personal health influences behaviour. | “Yes, sure, this is a big deal. Especially movement, this is the key for me. If I cannot take painkillers, I cannot move. This is just as it is.” (T8, Pos. 35) | — |
| **Intrinsic Will** | Behaviour is attributed to one’s own willpower or lack thereof. | “Well, I must learn to withstand. It doesn’t help when he now sits silently… I must be able to watch other people eat.” (T1, Pos. 35) | — |
| **Psychological Factors** | Perception that mental health or psychological processes play a role in behaviour. | “Um..., yes, for me it's a bit of a mental thing. If I'm not feeling well mentally, the scales don't go down. On the contrary, it goes up.” (T4, Pos. 45) | — |
| **Changes** | Realization of change in personal or others’ behaviour. | “That didn't really have any influence. (...) So what I had before, nothing changed, so to speak, but a lot of things changed through HAPpEN.” (T14, Pos. 62) | — |
| **Monetary Matters** | Financial considerations in relation to behaviour. | “My biggest fear in the beginning was that if I lose weight, I need new clothes, but I don’t have money for that.” (T17, Pos. 38) | Applies when monetary issues are mentioned in direct relation to behaviour. |
| **Special Circumstances (Death, Grief, Illness)** | Behaviour is explained by exceptional circumstances such as loss or illness. | “Well, I wasn't really involved for five months now. (...) Because we had a death in the family, that really threw me off track.” (T9, Pos. 77) | Must show causal relationship between the event and behaviour. |
| **Social Control** | Perceived social pressure influencing behaviour. | “My colleagues pay attention that I don’t eat the wrong things.” (T6, Pos. 9) | Distinguish from comparison and expectations from others. |
| **Habitualness** | Habitual behaviour as a factor. | “But if you don't do it every day, it will just fall asleep again at some point.” (T7, Pos. 40) | Distinguish from socialization: habitualness is learned in later life. |
| **Expectations of Others** | Behaviour is influenced by what others expect. | “Now we have vegetables in the fridge, but she expects something else, so I won’t use it. And I don’t cook for me alone.” (T16, Pos. 32) | Distinguish from social control and comparison. |
| **Individual Living Conditions** | Personal circumstances that influence behaviour. | “My husband is a hunter and often is out in the woods, I often join him. And my son also takes part in HAPpEN, that makes cooking easier.” (T10, Pos. 8) | — |
| **Socialization** | Influence of cultural or early life social learning. | “We grew up like that too. The fatter, the better because we must have something to give, that's what it used to be called.” (T1, Pos. 41) | Distinguish from habitualness: socialization refers to early, unconscious patterns. |
| **Personal Interest** | Personal preferences guiding behaviour. | “So occasionally we meet up for a swim, if the shift allows it. But I’d rather do nothing.” (T9, Pos. 27) | — |
| **Genetics** | Perceived genetic influence on behaviour or health. | “Intestinal disease, intestinal surgery, nutrition... it's contradictory. Oatmeal is good but can inflame the gut. Fear plays a very big role.” (T1, Pos. 61) | — |

Table S3: Coding Manual – **External Determinants**

| **Subcategory** / Sub-Subcategory | **Definition** | **Anchor Example** | **Coding Rule** |
| --- | --- | --- | --- |
| **Determinants** | External elements influencing behaviour. | — | Applies to all subcategories: distinguish from internal thought patterns. |
| **Genetics** | Non-changeable characteristics such as age, gender, family history. | “The genes. Both my grandparents on both sides are diabetics.” (T1, Pos. 55) | — |
| **Individual Lifestyle** | Behaviours like diet, alcohol, or tobacco use. | “We always buy a normal crate. My best friend wouldn’t change it now, so I just drink the regular one.” (T16, Pos. 28) | — |
| **Social Network and Support** | Social and communal embeddedness and support. | — | — |
| Motivation | Uplift or encouragement from others. | “We actually motivate each other when we do something together.” (T8, Pos. 53) | — |
| Tangible Support | Concrete support (e.g., money, joint plans). | “My mom gave me money to buy new pants.” (T16, Pos. 38) | — |
| Negative Network Influence | Negative environmental/social influence. | “Birthdays always have champagne, sweets, and cake. I used to always say yes.” (T3, Pos. 25) | — |
| Role Modelling | Seeing others as behavioural examples. | “Someone who has already lost 52 kilos said, ‘this helped me’ – that's helpful.” (T4, Pos. 35) | — |
| Social Belonging | Being part of a social unit. | “My mom sometimes calls to see if I want to go with her for a walk.” (T16, Pos. 28) | — |
| Practical Tips | Practical help or instructions. | “My son has a lot of equipment and gave me some instructions.” (T3, Pos. 37) | — |
| **Life and Work Conditions** | Meso-level conditions surrounding the individual. | “The framework conditions play a major role.” (T1, Pos. 57) | — |
| Food Availability / Infrastructure | External supply or infrastructure related to nutrition. | “Eating out is difficult; we consciously choose where we go.” (T3, Pos. 57) | — |
| Education | Formal/informal health-related knowledge. | “My husband always valued good food. In the choir, knowledge is less present.” (T3, Pos. 29) | Distinguish from socialization/habits: this is conscious knowledge. |
| Work | Influence of the working environment. | “I work in a residential home. We always have a sports session on Tuesdays and Saturdays.” (T16, Pos. 8) | — |
| Unemployment | *Did not appear in the data.* | — | — |
| Water and Sanitary Environment | *Did not appear in the data.* | — | — |
| Health Care System | *Did not appear in the data.* | — | — |
| Housing Conditions | *Did not appear in the data.* | — | — |
| **General Socioeconomic, Cultural, or Physical Environment** | Macro-level population-wide or subgroup influences. | “It's difficult in the countryside... paths aren't well developed, and my life is more important than being hit by a car.” (T9, Pos. 81) | Distinguish from specific factors. |

Table S4: Coding Manual – Relational Direction

| **Main Category / Subcategory** | **Definition** | **Anchor Example** | **Coding Rule** |
| --- | --- | --- | --- |
| **Relational Direction** | Perceived directionality of relational influence. | — | — |
| **Balanced** | Relationship perceived as mutual give-and-take. | “So, we both take it as a... psychological discussion group... it's a give and take.” (T1, Pos. 45) | — |
| **Outwards > Ego** | External actors influence the participant. | “At work, I was sceptical at first, but now I see it’s actually good.” (T16, Pos. 26) | Includes change processes or behaviour as reaction to others. |
| **Ego > Outwards** | The participant influences their environment. | “I am the initiator and have already tried to cook more health-consciously.” (T13, Pos. 25) | Includes change initiated by the participant. |

Table S5: Coding Manual – Network Relation

| **Main Category / Subcategory** | **Definition** | **Anchor Example (Translated)** | **Coding Rule** |
| --- | --- | --- | --- |
| **Network Relation** | Whether the behaviour is tied to a person from the social environment. | — | Distinguish from environmental conditions. Only apply if an interpersonal influence is mentioned. |
| **No Network Relation** | No social contact is relevant. | “No, I don’t speak with anyone about food or physical activity.” (T5, Pos. 29) | — |
| **Partner** | Behaviour is tied to a spouse/partner. | “I cook the healthy option with vegetables, and my husband eats without or with fewer.” (T1, Pos. 7) | Must mention behavioural change related to the partner. |
| **Parents** | Tied to a parent. | “I live with my dad and brother. We eat at my dad’s once a week.” (T4, Pos. 9) | Must mention behavioural change related to the parents. |
| **Further Family** | Extended family influence. | “One of my sisters was quite taken with this program.” (T15, Pos. 8) | Distinguish from parents, children, and spouse. |
| **Friends & Further Contacts** | Social contacts outside family or work. | “I run a choir. I talk about it with them.” (T3, Pos. 15) | Distinguish from colleagues. |
| **Children** | Tied to one’s children. | “The kids and I decide on leisure time activities.” (T5, Pos. 13) | Distinguish from other family members. |
| **Colleagues** | Tied to work colleagues. | “My colleagues tell me I cannot eat this treat because I take part in the program.” (T6, Pos. 13) | Distinguish from other social contacts. |
